# Identification of *Lagopus muta japonica* food plant resources in the Northern Japan Alps using DNA metabarcoding

**DOI:** 10.1371/journal.pone.0252632

**Published:** 2022-03-10

**Authors:** Taichi Fujii, Kaoru Ueno, Tomoyasu Shirako, Masatoshi Nakamura, Motoyasu Minami

**Affiliations:** 1 Bioscience and Biotechnology, Chubu University, Kasugai, Aichi, Japan; 2 Institute of Environmental Ecology, IDEA Consultants, Inc., Riemon, Yaizu, Shizuoka, Japan; Institute for Biological Research, University of Belgrade, SERBIA

## Abstract

DNA metabarcoding was employed to identify plant-derived food resources for the Japanese rock ptarmigan (*Lagopus muta japonica*), which is registered as a natural living monument in Japan, in the Northern Japanese Alps in Toyama Prefecture, Japan, in July to October, 2015–2018. DNA metabarcoding using high-throughput sequencing (HTS) of *rbcL* and ITS2 sequences from alpine plants found in ptarmigan fecal samples collected in the study area. The obtained sequences were analyzed using a combination of a constructed local database and the National Center for Biotechnology Information (NCBI) database, revealed that a total of 53 plant taxa were food plant resources for ptarmigans. Of these plant taxa, 49 could be assigned to species (92.5%), three to genus (5.7%), and one to family (1.9%). Of the 23 plant families identified from the 105 fecal samples collected, the dominant families throughout all collection periods were Ericaceae (99.0% of 105 fecal samples), followed by Rosaceae (42.9%), Apiaceae (35.2%), and Poaceae (21.0%). In all of the fecal samples examined, the most frequently encountered plant species were *Vaccinium ovalifolium* var. *ovalifolium* (69.5%), followed by *Empetrum nigrum* var. *japonicum* (68.6%), *Kalmia procumbens* (42.9%), *Tilingia ajanensis* (34.3%) and *V*. *uliginosum* var. *japonicum* (34.3%). A rarefaction analysis for each collection period in the study revealed that the food plant resources found in the study area ranged from a minimum of 87.0% in July to a maximum of 97.5% in September, and that 96.4% of the food plant taxa were found throughout the study period. The findings showed that DNA metabarcoding using HTS to construct a local database of *rbcL* and ITS2 sequences in conjunction with *rbcL* and ITS2 sequences deposited at the NCBI, as well as rarefaction analysis, are well suited to identifying the dominant food plants in the diet of Japanese rock ptarmigans. In the windswept alpine dwarf shrub community found in the study area, dominant taxa in the Ericaceae family were the major food plant s for Japanese rock ptarmigans from July to October. This plant community therefore needs to be conserved in order to protect the food resources of Japanese rock ptarmigans in the region.

## Introduction

Global climate change will alter the current distribution patterns of alpine biota, threatening many of the endemic species that are adapted to alpine ecosystems with extinction [1]. For endemic species whose distributions are restricted to the alpine meadow zone above the forest line, these habitats may shrink due to altitudinal shifts in forest tree species in response to climate warming [2,3]. In particular, there is concern that changes in the habitat of alpine flora due to global warming will result in the extinction of endangered animals that utilize endemic alpine plants that grow above the tree line in the alpine meadow zone as food resources. Consequently, a detailed understanding of the food resources of endangered animals in alpine environments is necessary for the effective conservation of these ecosystems.

The rock ptarmigan (*Lagopus muta*) is a medium-sized grouse that is widely distributed in the subarctic regions of Eurasia and North America [4]. The Japanese rock ptarmigan (*L*. *m*. *japonica*), a subspecies of the rock ptarmigan, is endemic to Japan and resides in the southernmost region of the rock ptarmigan’s habitat [4,5]. The Japanese rock ptarmigan is a relict species that remained on the Japanese archipelago after the last glacial ice age approximately 70,000 to 10,000 years ago [4]. The Japanese rock ptarmigan inhabits the alpine meadow zone, which is above the forest limit and is scattered with *Pinus pumila* at altitudes ranging from 2,000 to 3,000 m above sea level (a.s.l.) on the Japanese mainland [4,5], where it feeds mainly on the alpine plants found in the region. During the breeding season (April to May), the male occupies a territory of 0.015 to 0.072 km^2^ [6]. The female builds her nest at the base of *P*. *pumila* trees from June to July, and lays her eggs in July [4,7]. The female alone incubates the eggs and raises the chicks until October, at which time they become independent [4]. When their habitat (alpine meadow zone) is covered with snow (November to April), the Japanese rock ptarmigan migrates to the forest zone, returning to the alpine meadow zone as the snow begins to melt [8].

Although Japanese people have traditionally regarded the Japanese rock ptarmigan as sacred since ancient times, and have protected it from being captured or hunted, populations of this species on several mountains have either declined or become locally extinct since the 1930s [4]. In 1955, the Japanese rock ptarmigan was registered as a natural living monument in Japan because of its scarcity, and it is currently the focus of intensive conservation efforts [4]. Haneda et al. [9] estimated that the population of Japanese rock ptarmigan was approximately 3,000 individuals in the 1980s. Despite ongoing conservation efforts, by the early 2000s, the population of Japanese rock ptarmigans was estimated to number approximately 1,700 individuals [4]. The reasons for this decline are poorly understood; however, loss of alpine meadow vegetation and damage by Sika deer (*Cervus nippon*) are thought to be primarily responsible [10]. In addition, areas of alpine meadow vegetation have been damaged extensively by mountain climbers in the alpine belt [11], and more recently, concerns have been raised about changes in habitat due to climate change. It is predicted that the optimal habitat of this species will be reduced drastically from 2081 to 2100 and that the risk of future extinction is very high [6]. To address these issues, the restoration and management of alpine meadow flora are being carried out in various alpine areas in Japan [12,13]. However, none of these efforts have considered the impact of food plants utilized by the Japanese rock ptarmigan, primarily because few surveys of the food plants for this species have been conducted to date. In addition, breeding programs have been implemented in an attempt to increase the population; however, the mortality of chicks raised in captivity is high, likely due to inappropriate dietary composition [14,15]. For these reasons, a detailed understanding of the alpine meadow vegetation utilized by Japanese rock ptarmigans as a food source is important for the future conservation of this species [16].

Given this background, several methods have been employed to qualitatively or quantitatively elucidate the composition of the Japanese rock ptarmigan diet. Previous methods used to identify the food plant resources of this species have included stomach content analysis by dissecting birds [17,18], microscopic analysis of food plant residues in feces [19,20], direct observations of its foraging behavior [16], and DNA barcoding methods using feces [21,22]. While stomach content analysis by dissection is the most well established of these methods [17,18], the protected status of the Japanese rock ptarmigan in Japan means that such destructive methods cannot be employed. Although microscopic analysis of food plant residues in feces circumvents the need to sacrifice the animals, such methods have been shown to be relatively inaccurate. For example, in a study on fecal residues in L. m. pyrenaica in the French Pyrenees, the plant residues were highly degraded and could not be identified to species [19,20]. Consequently, the most common method for investigating the food preference of this species is by direct observations of its foraging behavior; however, this method requires long-term observations by numerous observers [16]. Further, the accuracy of direct observation methods is highly dependent upon specific training in plant species identification, which means that this method is neither objective nor reproducible. Direct observations can also stress the birds due to the presence of observers, and the alpine meadow vegetation can be damaged by trampling [16]. It is therefore necessary to establish a stress-free survey method that can be used to identify the food plant resources of the Japanese rock ptarmigan to the species level more rapidly than the previously reported survey methods [16–20].

The DNA barcoding method has been successfully applied to identify food residues in feces and gastric contents in wild animals [23–37]. For example, we previously conducted a study to identify food plants in the diet of Japanese rock ptarmigan in the Northern Alps of Japan using DNA barcoding methods [22]. In that study, the results of cloning experiments and a sequence analysis using a combination of a local database, which contained chloroplast *rbcL* (*rbcL*) sequences constructed from alpine plant species found in fecal samples collected in the study area, and sequences deposited at the National Center for Biotechnology Information (NCBI) for *rbcL* sequences from 73 alpine plant species in the study area revealed that there were a total of 26 food plant taxa, 22 of which could be identified to species, two to genus, and two to family [22]. Compared with previous studies on other groups, the use of DNA barcoding in conjunction with a local database and sequences deposited in the NCBI database in our previous study [22] enabled us to identify more plant species than was possible by observation of gastric contents alone [17,18], and almost the same number of plant species as using direct observations of foraging behavior, but in a shorter period of time[16]. However, the cloning method used for the short *rbcL* region in our previous study generated a limited amount of data [22]. For example, the rarefaction and extrapolation sampling curves revealed that the survey covered 89% of food plant candidates present in the study area [22]. The findings meant that our previous method may have underestimated the number of plant species foraged by ptarmigan, as only a limited number of DNA sequences could be obtained from one fecal sample using the cloning method, and not all of the food plant candidates in the study area were detected. Thus, there are major obstacles to identifying food plant residues in feces to species level and to capture all of the food plant candidates in an area using only cloned *rbcL* DNA sequences. However, DNA metabarcoding using high-throughput sequencing (HTS) can determine more DNA sequences at a higher level of sensitivity than the cloning method [23–37]. In addition, interspecific DNA polymorphisms in short *rbcL* region used in our previous studies could not be identified to species for several frequently foraged taxa e.g., Asteraceae, Vaccinium sp., and Rhododendron sp. [22]. However, given that most of the DNA in feces is typically degraded, obtaining long PCR fragments to improve the accuracy of species identification is difficult [38]. To solve these problems, the resolution of plant species identification by DNA barcoding can be improved by combining analyses of *rbcL* sequences, which have been deposited for many plants in DNA databanks, with analyses of the internal transcribed spacer 2 region (i.e. the region between 5.8S ribosomal RNA and 28S ribosomal RNA) of nuclear DNA (ITS2), as this latter region shows high levels of interspecific variability [39,40]. Consequently, because ITS2 has higher levels of interspecific variation than *rbcL* [41], ITS2 is generally considered to be better suited to species level identification by DNA barcoding than chloroplast DNA [42,43]. In addition, previous results obtained using a combination of a local database and the NCBI database were more accurate than queries of the NCBI database alone [22]. Other studies also showed that analyses performed using comprehensive local databases had relatively high levels of resolution [44,45]. Therefore, to ensure that the accuracy of plant species identification was higher than that obtained in our previous DNA barcoding studies [21,22], the NCBI database was used in conjunction with local databases containing *rbcL* and ITS2 sequences for alpine plant species found in fecal samples collected the study area.

Thus, this study identified the dietary components of a Japanese rock ptarmigan population by DNA metabarcoding using a local database containing alpine plant *rbcL* and ITS2 sequences that were obtained by HTS in conjunction with the same sequences deposited in the NCBI database. The *rbcL* and ITS2 sequences obtained from ptarmigan fecal samples collected in the area were then compared with the sequences in these databases to identify the food plants of the ptarmigan in the study area. Further, the effectiveness of metabarcoding using both *rbcL* and ITS2 was also evaluated and compared with separate analyses using either the *rbcL* or the ITS2 data. We used these methods to identify the major food plant resources for the Japanese rock ptarmigan during the brooding duration (July to October) in Japan’s Northern Alps, which is at the center of their distribution area. In addition, we examined the influence for each fecal sampling period on the variation in major food plant resources. In order to evaluate the completeness of our sampling efforts, a rarefaction analysis was conducted to estimate the percentage coverage of the number of identified food plant taxa relative to the number of fecal samples analyzed [46–49]. The advantages of the current DNA metabarcoding approach using a local database containing *rbcL* and ITS2 sequences in conjunction with the NCBI database were compared with previous studies on plant resource utilization in Japanese rock ptarmigan [16–18,22]. Finally, based on the obtained results, we discussed the conservation of alpine flora, which are utilized by Japanese rock ptarmigan as a food resource.

## Materials and methods

### Study area

**Fig 1:**
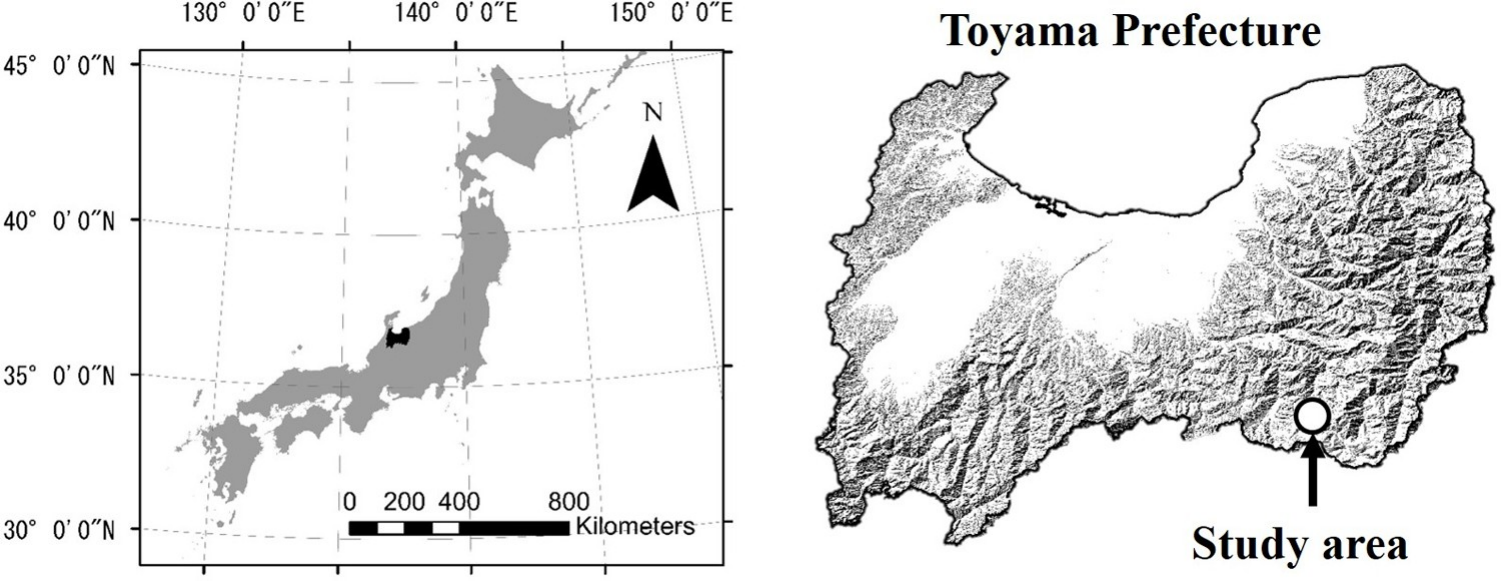
Location of the study area on Mt. Taro in Chubu-Sangaku National Park, at the western edge of the Northern Alps, Toyama Prefecture, Japan. The maps are reproduced from digital maps published by the Ministry of Land, Infrastructure, Transport and Tourism (https://nlftp.mlit.go.jp/ksj/gml/datalist/KsjTmplt-N03-v3_0.html) (left) and a digital elevation model (DEM; 10 m mesh) published by the Geospatial Information Authority of Japan (https://fgd.gsi.go.jp/download/menu.php) (right).

This study was conducted in full compliance with Japanese nature park laws and regulations, including obtaining a license from the Ministry of Environment of Japan to collect Japanese rock ptarmigan feces and plant species. The study was carried out at altitudes ranging from 2,324 to 2,373 m a.s.l. on and around the peak of Mt. Taro (36°26’50.1” N, 137°30’48.9” E, 2373 m a.s.l.), in Chubu-Sangaku National Park at the western end of Japan’s Northern Alps, Toyama Prefecture, Japan (Fig 1). In the study area (ca. 70 ha), the plant community on the northwest-facing slope was dominated by the species *Empetrum nigrum* var. *japonicum*, *Vaccinium uliginosum* var. *japonicum*, *Sasa kurilensis*, and *P*. *pumila*; and that on the southeast-facing slope was dominated by the species *Phyllodoce aleutica* and *Kalmia procumbens*. In addition, *Betula ermanii* and family Poaceae grew on the bare field along the wooden footpath, and *Nephrophyllidium crista*-*galli* subsp. *japonicum*, *Eriophorum vaginatum*, and *Juncus filiformis* grew in and around the small pools.

### Collection of fecal samples and plants

In the study area, typically three pairs of Japanese rock ptarmigans are observed every year. Since Japanese rock ptarmigans show no fear of humans, continuous monitoring is possible for collection of fecal samples. While mainly monitoring the covey, consisting of the adult female and her chicks, from July to October, 2015–2018, fecal samples that were visually confirmed to have been excreted were collected whenever possible during the study period (Table 1). The female is always in close proximity to her chicks, and, according to our observations, the plant species foraged by the female and her chicks were almost the same. Therefore, the feces of the female and her chicks were not considered separately, and a total of 116 fecal samples were collected. The collected fecal samples were stored at −20°C until total genomic DNA extraction. In order to construct the *rbcL* and ITS2 local databases, samples of 73 plant species were collected from within the study area on July 27, 2016 (Table 2). These plant specimens were stored under air-dried conditions in the presence of silica gel until total genomic DNA extraction.

**Table 1:** List of dates when fecal samples were collected, number of fecal samples used in the experiment, and number of fecal samples that were successfully DNA metabarcoded.

Feces collection period	Number of collected fecal samples	Number of fecal samples from which *rbcL* and ITS2 sequences were obtained
July 8–10, 2016	32	31
July 26–28, 2016	18	18
August 18–20, 2015	33	31
September 13–14, 2018	20	17
October 11–12, 2016	13	8
Total	116	105

**Table 2:** Local databases constructed from alpine plants in the study area.

Family name	Scientific name	Local database^1)^	
		*rbcL*	ITS2
Apiaceae	*Peucedanum multivittatum*	LC376994	LC554299
	*Tilingia ajanensis*	LC376976	LC554281
Aquifoliaceae	*Ilex sugerokii* var. *brevipedunculata*	LC377022	LC554325
Asparagaceae	*Maianthemum dilatatum* ^2)^	LC377033	KY908558
Asteraceae	*Anaphalis margaritacea* var. *margaritacea*	LC376983	LC554288
	*Arnica unalaschcensis* var. *tschonoskyi*	LC377026	LC554329
	*Artemisia sinanensis*	LC377038	LC554338
	*Cirsium otayae*	LC377005 ^a^	LC554310
	*Hieracium japonicum*	LC377028 ^a^	LC554331
	*Ixeridium dentatum* subsp. *kimuranum*	LC377017	LC554320
	*Solidago virgaurea* subsp. *asiatica*	LC376986 ^a^	LC554291
Betulaceae	*Betula ermanii*	LC377009	LC554314
Brassicaceae	*Cardamine nipponica*	LC377000	LC554305
Celastraceae	*Parnassia palustris*	LC377029	LC554332
Cornaceae	*Cornus canadensis* ^2)^	LC377034	MG218733
Cupressaceae	*Juniperus communis* var. *hondoensis*	LC376993	LC554298
Cyperaceae	*Carex blepharicarpa*	LC377013	LC554317
	*C*. *brunnescens*	LC377014	LC554318
	*C*. *pyrenaica* var. *altior*	LC377030	LC554333
	*C*. *nubigena*	LC377012	LC554316
	*Eriophorum vaginatum* ^2)^	LC377016	JX566737
Diapensiaceae	*Schizocodon soldanelloides f*. *alpinus*	LC377002	LC554307
Droseraceae	*Drosera rotundifolia*	LC377035	LC554336
Ericaceae	*Andromeda polifolia*	LC377023	LC554326
	*Arctous alpina*	LC376978	LC554283
	*Elliottia bracteata*	LC377027	LC554330
	*Empetrum nigrum* var. *japonicum*	LC377004	LC554309
	*Eubotryoides grayana* var. *parvifolia*	LC377019	LC554322
	*Gaultheria adenothrix*	LC377041	LC554341 ^d^
	*G*. *pyroloides*	LC377032	LC554335 ^d^
	*Kalmia procumbens*	LC376974	LC554279
	*Phyllodoce aleutica*	LC377042	LC554275 ^e^
	*P*. *nipponica*	LC376973	LC554278 ^e^
	*Rhododendron brachycarpum*	LC377021 ^b^	LC554324
	*R*. *tschonoskii* subsp. *trinerve*	LC377015 ^b^	LC554319
	*Vaccinium ovalifolium* var. *ovalifolium*	LC377020	LC554323 ^f^
	*V*. *shikokianum*	LC377040 ^c^	LC554340 ^f^
	*V*. *smallii* var. *smallii*	LC376970 ^c^	LC554276 ^f^
	*V*. *uliginosum* var. *japonicum*	LC376988 ^c^	LC554293 ^f^
	*V*. *vitis-idaea* ^3)^	KF163412	GU361898
Gentianaceae	*Gentiana makinoi*	LC376998	LC554303
	*G*. *nipponica*	LC376979	LC554284
	*G*. *thunbergii* var. *minor*	LC376971	LC554277
Hypericaceae	*Hypericum senanense* subsp. *mutiloides*	LC376985	LC554290
Juncaceae	*Juncus filiformis*	LC376984	LC554289
Lentibulariaceae	*Pinguicula vulgaris* var. *macroceras*	LC376987	LC554292
Melanthiaceae	*Helonias orientalis* ^4)^	LC377011	No data
	*Veratrum stamineum* var. *stamineum*	LC376981	LC554286
Menyanthaceae	*Nephrophyllidium crista-galli* subsp. *japonicum*	LC376991	LC554296
Nartheciaceae	*Aletris foliata*	LC376999	LC554304
	*Narthecium asiaticum*	LC377024	LC554327
Orchidaceae	*Dactylorhiza aristata*	LC376997	LC554302
	*Platanthera tipuloides* subsp. *nipponica*	LC377025	LC554328
Orobanchaceae	*Pedicularis chamissonis* var. *japonica*	LC376995	LC554300
	*P*. *yezoensis*	LC376977	LC554282
Pinaceae	*Abies mariesii*	LC377010	LC554315
	*Pinus pumila*	LC376992	LC554297
Plantaginaceae	*Veronica nipponica*	LC377031	LC554334
Poaceae	*Calamagrostis longiseta*	LC376990	LC554295
	*Moliniopsis japonica*	LC377039	LC554339
	*Sasa kurilensis* ^4)^	LC376972	No data
Polygonaceae	*Persicaria weyrichii* var. *weyrichii*	LC377006	LC554311
	*Rumex alpestris* subsp. *lapponicus*	LC377008	LC554313
Polytrichaceae	*Polytrichum juniperinum* ^2)^	LC377037	MF180404
Ranunculaceae	*Anemone narcissiflora* subsp. *nipponica*	LC376982	LC554287
	*Coptis trifoliolata*	LC376975	LC554280
	*Ranunculus acris* subsp. *nipponicus*	LC376996	LC554301
Rosaceae	*Aruncus dioicus* var. *kamtschaticus*	LC377018	LC554321
	*Potentilla matsumurae*	LC377001	LC554306
	*Sieversia pentapetala*	LC377003	LC554308
	*Sorbus commixta*	LC376989	LC554294
Sapindaceae	*Acer tschonoskii*	LC377007	LC554312
Tofieldiaceae	*Triantha japonica*	LC377036	LC554337
Xanthorrhoeaceae	*Hemerocallis dumortieri* var. *esculenta*	LC376980	LC554285

1) Accession numbers with the letters LC were deposited at DDBJ by the authors. Other accession numbers are quoted from NCBI. The same superscripted letters (a to f) indicate the same sequence.

2) Since ITS2 was not amplified in this study, DNA sequences of ITS2 registered in NCBI are given.

3) *Vaccinium vitis-idaea* was not collected in this study; DNA sequences for *rbcL* and ITS2 that have been deposited in the NCBI database are given.

4) The ITS2 product could not be amplified in this study and the ITS2 sequences are not registered in the NCBI database.

### Construction of *rbcL* and ITS2 local databases for the fecal samples collection area

The local *rbcL* database was constructed using the methods that we described previously [22]. Since the *rbcL* sequence is haploid, its DNA sequence can be determined by the direct sequencing method. Total DNA was extracted from air-dried leaves (ca. 0.8 cm2) of each alpine plant species using a DNeasy Plant Mini Kit (QIAGEN, Hilden, Germany) and purified using a GENECLEAN SPIN Kit (MP Biomedicals, CA, USA). PCR reactions were performed in reaction mixtures of 50 μl containing 1.25 units of MightyAmp DNA Polymerase Ver. 2 (Takara Bio Inc., Kusatsu, Japan) and 0.32 μM of each primer, according to the manufacturer’s instructions. Primers flanking the *rbcL* region were F3 (5′-TATCTTGGCAGCATTCCGAGTAACTCC-3′) and R3 (5′-GATTCGCAGATCCTCCAGACGTAGAGC-3′) [50]. PCR amplification was performed using a DNA Thermal Cycler (GeneAmp PCR System 9700, Applied Biosystems, CA) using an initial denaturation step of 98°C for 2 min, followed by 30 cycles of denaturation at 98°C for 10 s, annealing at 60°C for 15 s, and extension at 68°C for 20 s. The PCR products were prepared for sequencing using a NucleoSpin Plasmid QuickPure kit (Macherey-Nagel, Germany), as described by the manufacturer, and the purified PCR products were subjected to direct sequencing using a DTCS Quick Start kit (Beckman Coulter, CA) and the same primers used for PCR on an automatic sequencer (CEQ 2000XL, Beckman Coulter). On the other hand, since ITS2 is nuclear DNA, it may contain heterogeneous sequences within the same plant species. Therefore, using the same DNA samples that were used to construct the local *rbcL* database, the DNA sequencing of ITS2 was performed by HTS using the amplicon sequence method. Since amplicon sequencing by HTS generally decreases nucleotide diversity, adjacent DNA sequences are misrecognized on the flow cell and sequencing quality scores are reduced [51]. Therefore, in order to increase nucleotide diversity, we used frame-shifting primers [51] for the initial PCR (S1 Table). The initial PCRs of the ITS2 region were performed in reaction mixtures of 12.0 μl containing 6.0 μl of KAPA HiFi (Kapa Biosystems, Wilmington, MA, USA), 0.7 μl of each 10 μM primer mix (S1 Table), 2.0 μl of template DNA for measuring the DNA concentration with the Qubit dsDNA HS Assay (Thermo Fisher Scientific, Waltham, MA) (normalized to 5 ng/mL for quality control), and 3.3 μl of dH2O. The initial PCR of ITS2 was performed using the following conditions: initial denaturation at 95°C for 3 min, followed by 35 cycles of denaturation at 98°C for 20 s, annealing at 55°C for 15 s, and extension at 72°C for 30 s, with final extension at 72°C for 5 min. The initial PCR products of ITS2 were purified with an Agencourt AMPure XP kit (Beckman Coulter, Fullerton, CA, USA). To construct the DNA libraries using the second PCR, the i7 and i5 indexes (Illumina, San Diego, CA, USA) for identifying each sample, and P5 and P7 adapters (Illumina) for Miseq sequencing, were ligated to the purified initial PCR products. The second PCRs of ITS2 were performed in reaction mixtures of 24.0 μl with 12.0 μl of KAPA HiFi (Kapa Biosystems), 2.8 μl of 10 μM forward and reverse index primers (S1 Table), 2.0 μl of 1/10 initial PCR products, and 4.4 μl of dH2O. The second PCRs of ITS2 were performed under the following conditions: initial denaturation at 95°C for 3 min, followed by 12 cycles of denaturation at 98°C for 20 s and extension at 72°C for 15 s, with final extension at 72°C for 5 min. The DNA libraries of ITS2 were purified with an Agencourt AMPure XP kit (Beckman Coulter) and sequenced using a MiSeq Reagent Kit v3 (600-cycle format; Illumina) with an Illumina MiSeq sequencer at IDEA Consultants, Inc., following the manufacturer’s protocol. When the same plant species had multiple ITS2 DNA sequences, the DNA sequence with the highest number of reads was adopted as the DNA sequence for the ITS2 local database and was registered at the DNA databank of Japan (DDBJ) to avoid the risk of introducing contamination from other plant-derived DNA sequences by field sampling (detailed accession numbers of each *rbcL* and ITS2 sequence are given in Table 2). The DNA sequences for plant species that could not be amplified successfully by PCR were obtained from the NCBI database. However, to ensure identification accuracy, the DNA sequences for plant species containing Ns (unknown nucleotides) were excluded from our analysis. We constructed our local *rbcL* and ITS2 databases using the makeblastdb function implemented in NCBI BLAST+ version 2.6.0+ [52].

### DNA metabarcoding of *rbcL* and ITS2

Total DNA was isolated from dry feces (ca. 2.9–59.2 mg) using a DNeasy Plant Mini Kit (Qiagen, Hilden, Germany) and purified using a Geneclean Spin Kit (MP-Biomedicals, Santa Ana, CA, USA). PCR amplification and *rbcL* amplicon sequencing by HTS was performed using the DNA metabarcoding primer for *rbcL* (S1 Table) and the same reaction mixture, conditions and instruments that were used for constructing the local ITS2 database, except that the annealing temperature used for *rbcL* was 56°C instead of 55°C in the initial PCR. On the other hand, PCR amplification and ITS2 amplicon sequencing by HTS was performed using the same reaction mixture, conditions and instruments that were used to construct the local ITS2 database.

### Sequence data analysis

Since the demultiplexing function of the Illumina MiSeq sequencer (Illumina) does not evaluate the sequencing quality scores of the i7 and i5 index sequences, the default FASTQ files provided by the Illumina MiSeq sequencer (Illumina) often contain miss-tagged sequences [53]. To solve this problem, instead of using the default demultiplexing function of the Illumina MiSeq sequencer (Illumina), the raw MiSeq data were converted into FASTQ files using the bcl2fastq v2.18 program (Illumina), and the FASTQ files were then demultiplexed using the clsplitseq function implemented in Claident [54]. In general, the demultiplexed FASTQ files generated by the MiSeq sequencer are analyzed using either the amplicon sequence variant (ASV) and operational taxonomic unit (OTU) approach [55]. In this study, the demultiplexed FASTQ files were analyzed using the ASV method implemented in the DADA2 v1.10.1 package [55], with the statistical software R version 3.5.3 [56]. ASV can discriminate between DNA sequences that differ by a single nucleotide, since the original DNA sequence can be estimated even if it contains PCR amplification or sequencing errors [55]. At the quality filtering process, we manually checked the quality score distribution using the plotQualityProfile function implemented in DADA2 [55], and then trimmed the forward and reverse directions of both *rbcL* and ITS2 sequences by 200 and 250 bases, respectively, using the filterAndTrim function. After trimming, the forward and reverse sequences were combined using the mergePairs function of DADA2 [55], and chimeric DNA sequences were removed using the removeBimeraDenovo function of DADA2 [55]. Low-frequency ASVs, i.e., less than 1.0% of the total number of sequences, in each fecal sample were excluded from the DNA metabarcoding analysis. To normalize the number of DNA sequences in each *rbcL* and ITS2, rarefaction curves were calculated with the rarecurve function of the vegan package ver.2.5–5 [57] in R [56]. Using the calculated rarefaction curves, 1,000 DNA sequences in each *rbcL* and ITS2 were normalized for each fecal sample using the rrarefy function of the vegan package [57] to determine the ASVs for plant species identification.

### Homology search and identification of food plant resources

**Fig 2:**
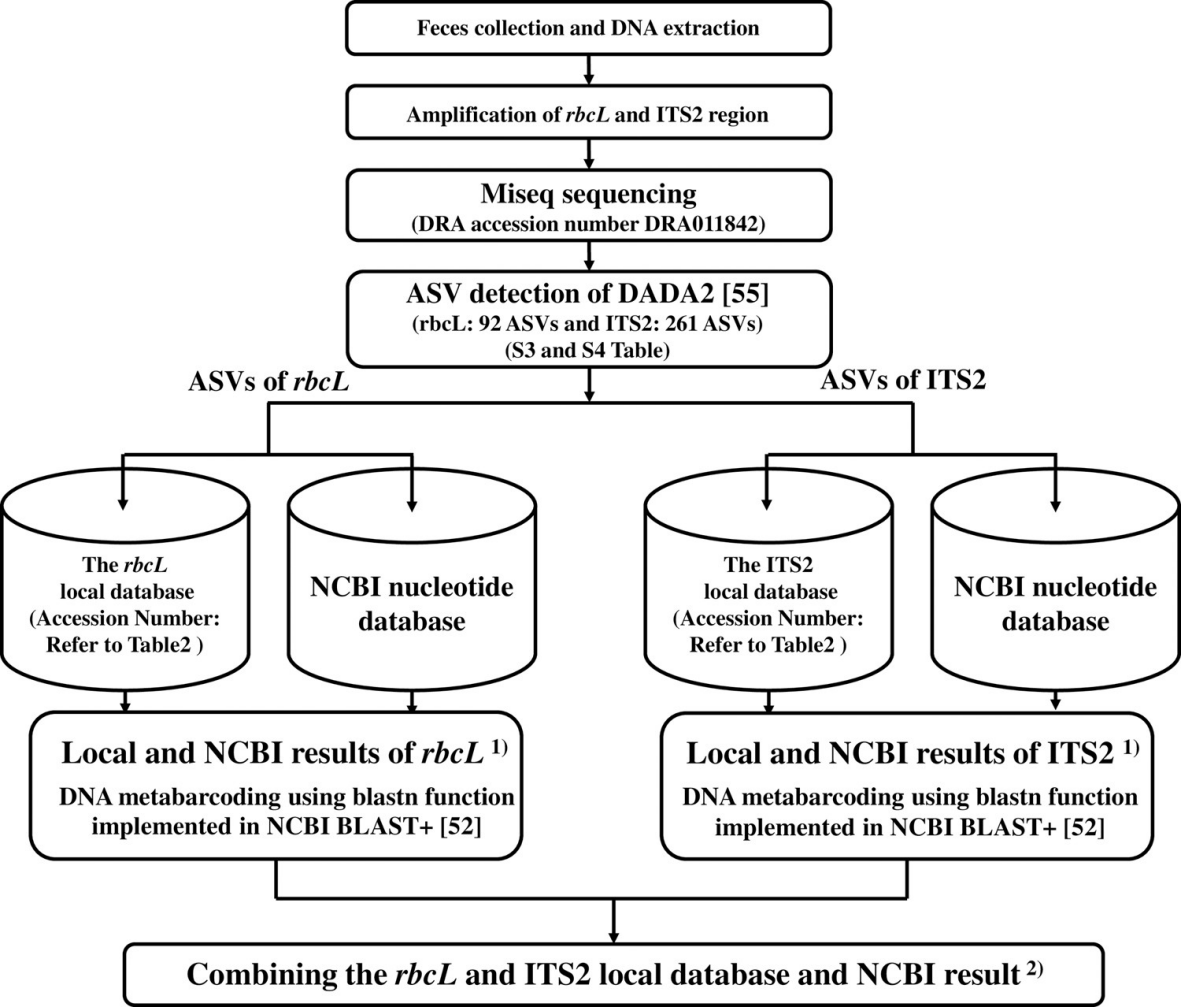
Flowchart of process used to identify food plant resources of Japanese rock ptarmigan in this study.

A flowchart of the method used in this study to identify the food plant resources of Japanese rock ptarmigans using DNA metabarcoding is shown in Fig 2. In this study, only fecal samples in which both *rbcL* and ITS2 could be PCR amplified were used as food plant identification samples. To identify food plants, homology searches were performed by comparing the ASVs obtained from the fecal samples against the DNA sequences for *rbcL* and ITS2 in our local database and all of the published sequences deposited in the NCBI database using the blastn function implemented in NCBI BLAST+ version 2.6.0+ [52]. To ensure that the accuracy of the identification was sufficiently robust, DNA sequences for *rbcL* and ITS2 with a homology of less than 98% and a bitscore of less than 300 were excluded from further analysis [22]. For both *rbcL* and ITS sequences, if the local database homology score was higher than that for the NCBI database, or if the homology scores obtained from the local database and NCBI were the same, the plant species identified by the local database was adopted. On the other hand, if the NCBI homology score was higher than that for the local database, the NCBI plant species was adopted. However, some of the plant species retrieved from the NCBI database did not grow in this study area in the Northern Japan Alps. Therefore, of the plant species that were from the NCBI database, we adopted only those that were confirmed to grow in the Japanese rock ptarmigan habitat in and around this study area by referring to the flora of the Northern Japan Alps [58–66]. In the event that a given sequence was identified as belonging to two or more taxa with the same score, that sequence was assigned to the highest taxonomic level that included both of those taxa [22]. As a result, some of the plant taxa identified using the local database were assigned to the rank of genus and others were assigned to family. On the other hand, some plant taxa identified using the NCBI database were assigned to the rank of genus or family, but others could not be identified to family level as multiple families. Eventually, we compared the taxonomic levels identified by *rbcL* or ITS2, and selected the lower taxonomic level. The accuracy of species identification was calculated by dividing the number of plant taxa identified to the species level by the total number of plant taxa.

1. Homology searches were performed by comparing the DNA sequences obtained from the fecal samples against the sequences in our *rbcL* and ITS2 local database and the NCBI database using the blastn function implemented in NCBI BLAST+ version 2.6.0+ [52]. To ensure that the accuracy of the identification was sufficiently robust, DNA sequences of *rbcL* and ITS2 with a homology of less than 98% and bitscore less than 300 were excluded from further analysis [22]. We compared the homology data obtained using the local database and NCBI, and selected the sequences that showed the highest homology as the local and NCBI results.
2. We compared the taxonomic levels identified by the local and NCBI databases based on *rbcL* and ITS2 sequences, and selected the lowest taxonomic level.

### Statistical analysis

The number of plant taxa per fecal sample identified using either the local database or the NCBI database for *rbcL* sequences, either the local database or the NCBI database for ITS2 sequences, and a combination of the *rbcL* and ITS2 local databases and the NCBI results were compared by the Steel-Dwass test (P<0.05) using the pSDCFlig function of the NSM3 package ver. 1.16 [67] in R. In addition, we calculated the percentage coverage as the number of identified food plant taxa relative to the number of analyzed fecal samples and estimated the asymptotic Shannon diversity index by rarefaction analysis using the iNEXT package ver. 2.0.20 [48,49,68] in R.

## Results

### Local database construction

Seventy-three alpine plant specimens, consisting of 32 families and 61 genera, were collected from the study area (Table 2) and used to construct the local *rbcL* and ITS2 databases. The *rbcL* and ITS2 sequences determined for the plant specimens were deposited in the NCBI database (see Table 2 for NCBI accession numbers). Our previous study showed that *Vaccinium vitis-idaea* was identified from the fecal samples of Japanese rock ptarmigans in our study area [21,22]; however, we were unable to collect this plant species. Therefore, for this plant species, we adopted the *rbcL* (KF163412) and ITS2 (GU361898) sequences that are registered in the NCBI database (Table 2). In this study, we included *V*. *vitis-idaea* in both of the local *rbcL* and ITS2 databases to give a total of 74 species in each database. The *rbcL* sequences could be determined for all 74 plant species (Table 2). Some taxa could only be identified to the genus or family level due to having the same *rbcL* sequences; three species in family Asteraceae (*Cirsium* *otayae*, *Hieracium japonicum*, and *Solidago virgaurea* subsp. *asiatica*), two species in genus *Rhododendron* (*R*. *brachycarpum* and *R*. *tschonoskii* subsp. *trinerve*), and three species in genus *Vaccinium* (*V*. *shikokianum*, *V*. *smallii* var. *smallii*, and *V*. *uliginosum* var. *japonicum*) [22]. On the other hand, 68/74 plant species could be identified to species using the ITS2 sequences (Table 2). For the four out of six ITS2 sequences that could not be amplified in this study, the sequences used in our local database of ITS2 sequences were obtained from NCBI (*Cornus canadensis*, MG218733, accession no.; *E*. *vaginatum*, JX566737; *Maianthemum dilatatum*, KY908558; and *Polytrichum juniperinum*, MF180404). The ITS2 sequences for the remaining two plant species (*Helonias orientalis* and *S*. *kurilensis*) were not registered in the NCBI database. In addition, two species in genus *Gaultheria* (*G*. *adenothrix* and *G*. *pyroloides*), two species in genus *Phyllodoce* (*P*. *aleutica* and *P*. *nipponica*), and four species in genus *Vaccinium* (*V*. *ovalifolium* var. *ovalifolium*, *V*. *shikokianum*, *V*. *smallii* var. *smallii*, and *V*. *uliginosum* var. *japonicum*) had the same ITS2 sequences. Finally, by combining the *rbcL* and ITS2 local databases, we constructed a local database containing sequences for 71 species and one genus (genus *Vaccinium*: including *V*. *shikokianum*, *V*. *smallii* var. *smallii*, and *V*. *uliginosum* var. *japonicum*) (Table 2).

### Sequence data processing

Of the 116 fecal samples collected, both *rbcL* and ITS2 were successfully amplified from 105 fecal samples (Table 1). Sequencing of the *rbcL* and ITS2 regions yielded a total of 6,030,040 and 2,307,861 DNA sequences (after preliminary quality filtering and removing chimeric sequences) with a mean of 57,429 and 21,980 DNA sequences per fecal sample, respectively (S2 Table). In this study, the *rbcL* and ITS2 sequence data were normalized to 1,000 DNA sequences, and ASVs were identified using 105,000 DNA sequences for each gene region (i.e., *rbcL* and ITS). A total of 92 and 261 ASVs were distinguished from each set of the 105,000 DNA sequences for *rbcL* and ITS, respectively (S3 and S4 Tables). All nucleotide sequence data were deposited in the DDBJ database (DRA accession number DRA011842). Based on the results obtained using either the local *rbcL* database or the local ITS2 databases, the number of ASVs with more than 98% homology were 64 out of 92 ASVs (69.6%) for *rbcL* and 121 out of 261 ASVs (46.4%) for ITS2, respectively (S3 and S4 Tables). Based on the results obtained using only the NCBI database, the number of ASVs with more than 98% homology were 47 out of 92 ASVs (51.1%) for *rbcL* and 167 out of 261 ASVs (64.0%) for ITS2, respectively (S3 and S4 Tables). Based on the results obtained by combining the *rbcL* and ITS2 local databases with the NCBI database, the number of ASVs with more than 98% homology was 62 out of 92 ASVs (67.4%) for *rbcL* and 149 (57.1%) out of 261 ASVs for ITS2, respectively (S3 and S4 Tables).

### Identification of food plants resources

The number and accuracy of plant taxa identified using the local database and the NCBI database for both *rbcL* and ITS2 are summarized in Table 3 (for detailed results of the homology searches, see S5 to S7 Tables). The homology results for the food plant taxa obtained using only the *rbcL* database are as follows. The local database identified a total of 36 plant taxa; 34 were identified to species (94.4% of 36 plant taxa), one to genus (2.8%), and one to family (2.8%). Analysis using the NCBI database identified a total of 34 plant taxa; 26 were identified to species (76.5% of 34 plant taxa), four to genus (11.8%), and three to family (8.8%). One taxon (2.9%) could not be identified to family level as multiple families were identified with the same score from one sequence. The combined results using the local database and the NCBI database identified a total of 40 plant taxa; 36 were identified to species (90.0% of 40 plant taxa), three to genus (7.5%), and one to family (2.5%). On the other hand, the homology results for the food plant taxa obtained using only the ITS2 database are as follows (Table 3). The local database identified a total of 29 plant taxa; 26 were identified to species (89.7% of 29 plant taxa) and three to genus (10.3%). The NCBI results identified a total of 31 plant taxa and all 31 were identified to species (100% of 31 plant taxa). The combined results using the local database and the NCBI database identified a total of 33 plant taxa; 30 were identified to species (90.9% of 33 plant taxa) and three to genus (9.1%). Compared to using only the local database or the NCBI database, the combined use of both databases for analyzing both of the *rbcL* and the ITS2 regions resulted in an increase in the accuracy of plant species identification, and the number of plant taxa identified was also higher (Table 3). In addition, combining the *rbcL* and the ITS2 databases improved the accuracy of plant species identification compared to analyses for each region alone (Table 3). The highest number of plant taxa identified per fecal sample was obtained by combining the *rbcL* and ITS2 local databases and the NCBI results (median 5, interquartile ranges 3–6), followed by the local databases and NCBI results for *rbcL* (4, 3–5), and the local databases and NCBI results for ITS2 (3, 2–4) (Steel-Dwass test, P<0.05) (S1 Fig). Among all of the combinations tested, the results obtained using a combination of all three databases using both *rbcL* and ITS2 data showed the highest accuracy for plant species identification for the two DNA barcoding regions, i.e., a total of 53 plant taxa were identified; 49 to species (92.5% of 53 plant taxa), three to genus (5.7%), and one to family (1.9%) (Table 3).

**Table 3:** Number of identified plant taxa and accuracy of identification based on *rbcL* and ITS2 sequences in different databases.

DNA barcode regions	Level of identification	Local database	NCBI database	Local and NCBI databases
*rbcL*	Species	34 (94.4%)	26 (76.5%)	36 (90.0%)
	Genus	1 (2.8%)	4 (11.8%)	3 (7.5%)
	Family	1 (2.8%)	3 (8.8%)	1 (2.5%)
	Not identified to family	0 (0.0%)	1 (2.9%)	0 (0.0%)
	Total identified taxa	36	34	40
ITS2	Species	26 (89.7%)	31 (100.0%)	30 (90.9%)
	Genus	3 (10.3%)	0 (0.0%)	3 (9.1%)
	Family	0 (0.0%)	0 (0.0%)	0 (0.0%)
	Not identified to family	0 (0.0%)	0 (0.0%)	0 (0.0%)
	Total identified taxa	29	31	33
*rbcL* and ITS2	Species	45 (90.0%)	40 (90.9%)	49 (92.5%)
	Genus	3 (6.0%)	3 (6.8%)	3 (5.7%)
	Family	2 (4.0%)	1 (2.3%)	1 (1.9%)
	Not identified to family	0 (0.0%)	0 (0.0%)	0 (0.0%)
	Total identified taxa	50	44	53

For detailed results of homology searches obtained for each database, see S5 to S7 Tables.

**Fig 3:**
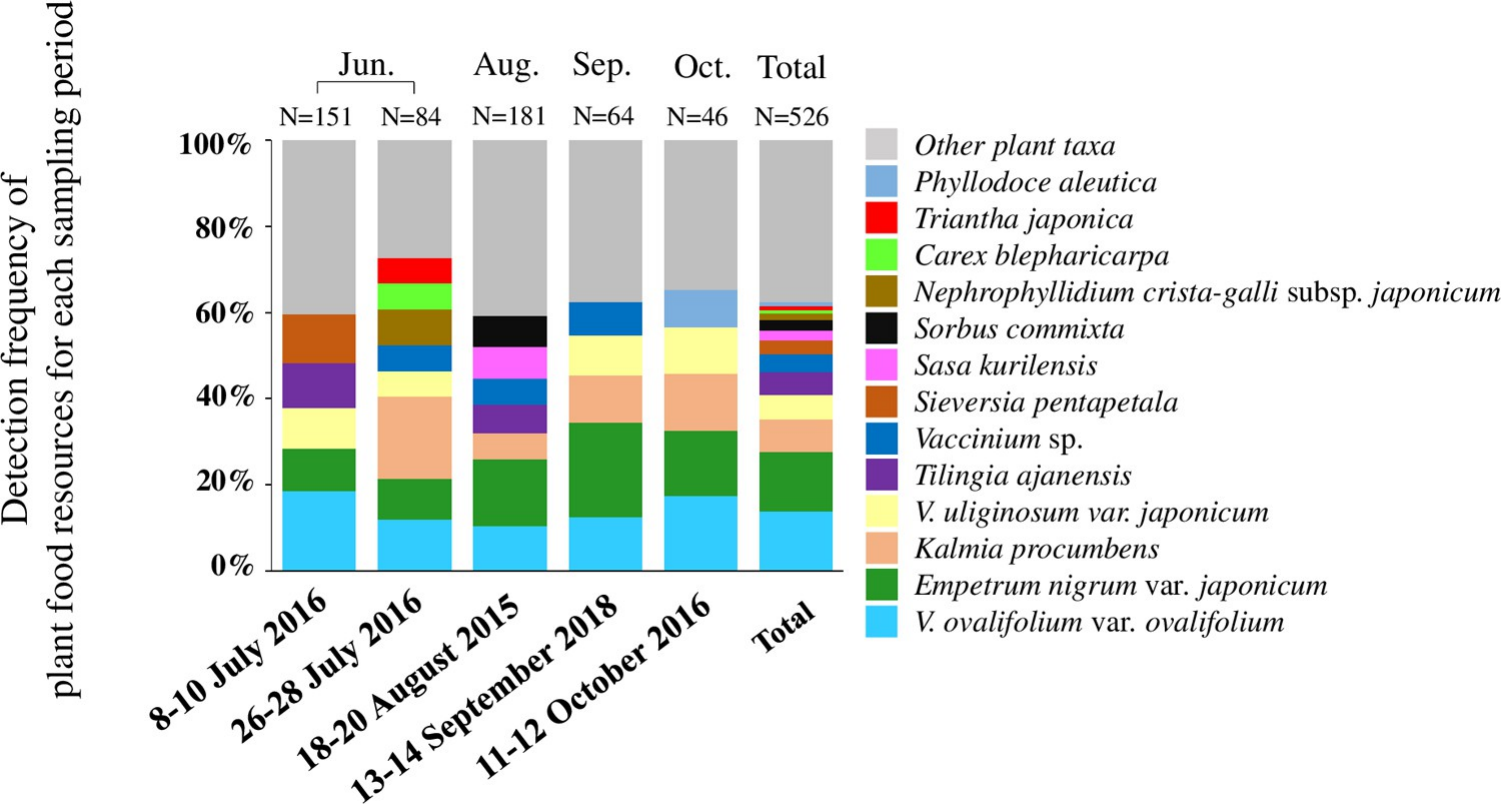
Intra-annual changes in major food plant taxa. The dominant plant species in each of the five fecal sampling periods are shown. N indicates the total number of food plant taxa for each fecal sampling period.

The homology results for the food-plant taxa obtained using a combination of local database and NCBI searches for both *rbcL* and ITS2 are summarized in Table 4. Of the 23 plant families identified using a combination of the local database and the NCBI database for both *rbcL* and ITS2 sequences, the dominant families throughout all collection periods were Ericaceae (99.0% of 105 fecal samples), followed by Rosaceae (42.9%), Apiaceae (35.2%), and Poaceae (21.0%) (S8 Table). In all of the fecal samples examined, the dominant food plants (more than 30% of 105 fecal samples) were *V*. *ovalifolium* var. *ovalifolium* (69.5%), followed by *E*. *nigrum* var. *japonicum* (68.6%), *K*. *procumbens* (42.9%), *Tilingia ajanensis* (34.3%) and *V*. *uliginosum var*. *japonicum* (34.3%) (Table 4). Remarkably, *V*. *ovalifolium* var. *ovalifolium* and *E*. *nigrum* var. *japonicum* were foraged throughout the study period, indicating that two plant taxa were the most important food resources in the study area (Fig 3). The subdominant plants (i.e., more than 10% to less than 30% of 105 fecal samples) were *Sieversia pentapetala* (29.5%), *Vaccinium* sp. (*V*. *ovalifolium* var. *ovalifolium*, *V*. *shikokianum*, *V*. *smallii* var. *smallii*, and *V*. *uliginosum* var. *japonicum*) (22.9%), *Sorbus commixta* (15.2%), *S*. *kurilensis* (14.3%), *P*. *aleutica* (13.3%), *V*. *vitis-idaea* (13.3%), *B*. *ermanii* (11.4%), *N*. *crista-galli* subsp. *japonicum* (11.4%) and *P*. *juniperinum* (11.4%) (Table 4). Of the subdominant plants, there was abundant foraging of *S*. *pentapetala* in July, and *S*. *commixta* and *S*. *kurilensis* in August (Fig 3 and Table 4). The estimated asymptotic Shannon index diversity varied over the study period; the diversity of foraged plant taxa tended to be higher from July to August (20.1–25.7) than from September to October (12.9–14.4) (Table 5). Rarefaction analysis of each collection period revealed that this study covered a minimum of 87.0% in July to a maximum of 97.5% in September of the food plant resources found in the study area, and 96.4% of the food plant taxa were covered throughout the entire study period (Table 5).

**Table 4:** Food plant taxa identified using DNA metabarcoding with *rbcL* and ITS2.

Family name	Scientific name	Database	Published previously in the literature	No. of fecal samples						Detection frequency per 105 fecal samples
				July 8–10, 2016 (n = 31)	July 26–28, 2016 (n = 18)	August 18–20, 2015 (n = 31)	September 13–14, 2018 (n = 17)	October 11–12, 2016 (n = 8)	Total (n = 105)	
Apiaceae	*Peucedanum multivittatum*	*rbcL* and ITS2	Yes	10	-	-	-	-	10	9.5%
	*Tilingia ajanensis*	*rbcL* and ITS2	Yes	16	4	12	4	-	36	34.3%
Aquifoliaceae	*Ilex sugerokii* var. *brevipedunculata*									
Asparagaceae	*Maianthemum dilatatum*		Yes							
Asteraceae	Asteraceae (*Cirsium otayae*, *Hieracium japonicum*, *Solidago virgaurea* subsp. *asiatica*)	*rbcL*	No	-	-	-	-	2	2	1.9%
	*Anaphalis margaritacea* var. *margaritacea*									
	*Arnica unalaschcensis* var. *tschonoskyi*		Yes							
	*Artemisia sinanensis*									
	*Cirsium otayae*									
	*Hieracium japonicum*									
	*Ixeridium dentatum* subsp. *kimuranum*	*rbcL* and ITS2	No	4	1	1	1	1	8	7.6%
	*Solidago virgaurea* subsp. *asiatica*	ITS2	No	-	-	1	-	-	1	1.0%
	*Taraxacum officinale*	ITS2	No	1	-	-	-	-	1	1.0%
Betulaceae	*Alnus hirsuta var*. *sibirica*	ITS2	No	1	-	-	-	-	1	1.0%
	*Betula ermanii*	*rbcL* and ITS2	Yes	4	-	2	4	2	12	11.4%
Brassicaceae	*Cardamine nipponica*									
Celastraceae	*Parnassia palustris*									
Cephaloziaceae	*Alobiellopsis parvifolia*	ITS2	No	1	-	-	-	-	1	1.0%
Cornaceae	*Cornus canadensis*	*rbcL*	No	-	-	4	-	2	6	5.7%
Cupressaceae	*Juniperus communis* var. *hondoensis*	*rbcL* and ITS2	No	1	1	-	-	-	2	1.9%
Cyperaceae	*Carex blepharicarpa*	ITS2	No	3	5	1	-	-	9	8.6%
	*C*. *brunnescens*									
	*C*. *oxyandra*	ITS2	No	-	-	1	-	-	1	1.0%
	*C*. *pyrenaica* var. *altior*	ITS2	No	-	1	-	-	-	1	1.0%
	*C*. *nubigena*									
	*Eriophorum vaginatum*									
Diapensiaceae	*Schizocodon soldanelloides* f. *alpinus*	*rbcL*	Yes	1	-	1	-	-	2	1.9%
Droseraceae	*Drosera rotundifolia*									
Ericaceae	*Andromeda polifolia*	*rbcL* and ITS2	No	-	3	-	-	-	3	2.9%
	*Arctous alpina*		Yes							
	*Elliottia bracteata*	*rbcL* and ITS2	Yes	1	-	1	-	-	2	1.9%
	*Empetrum nigrum* var. *japonicum*	*rbcL* and ITS2	Yes	15	8	28	14	7	72	68.6%
	*Epigaea asiatica*	*rbcL*	No	-	1	-	-	-	1	1.0%
	*Eubotryoides grayana* var. *parvifolia*									
	*Gaultheria adenothrix*	*rbcL*	No	-	-	-	3	1	4	3.8%
	*G*. *pyroloides*	*rbcL*	Yes	-	-	3	-	1	4	3.8%
	*Kalmia procumbens*	*rbcL* and ITS2	Yes	5	16	11	7	6	45	42.9%
	*Phyllodoce* sp. (*P*. *aleutica*, *P*. *nipponica*)	ITS2	Yes	1	-	1	-	-	2	1.9%
	*P*. *aleutica*	*rbcL*	Yes	4	-	4	2	4	14	13.3%
	*P*. *nipponica*	*rbcL*	Yes	1	1	1	-	-	4	2.9%
	*Rhododendron brachycarpum*									
	*R*. *tschonoskii* subsp. *trinerve*									
	*Vaccinium* sp. (*V*. *shikokianum*, *V*. *smallii* var. *smallii*, *V*. *uliginosum* var. *japonicum*)	*rbcL* and ITS2	Yes	3	5	11	5	-	24	22.9%
	*Vaccinium hirtum*	ITS2	No	-	-	1	-	-	1	1.0%
	*V*. *ovalifolium* var. *ovalifolium*	*rbcL*	Yes	28	10	19	8	8	73	69.5%
	*V*. *shikokianum*		Yes							
	*V*. *smallii* var. *smallii*		Yes							
	*V*. *uliginosum* var. *japonicum*		Yes	14	5	6	6	5	36	34.3%
	*V*. *vitis-idaea*	*rbcL* and ITS2	Yes	2	2	5	3	2	14	13.3%
Fabaceae	*Astragalus sinicus*	ITS2	No	1	-	-	-	-	1	1.0%
Gentianaceae	*Gentiana makinoi*									
	*G*. *nipponica*	*rbcL*	No	-	-	1	-	-	1	1.0%
	*G*. *thunbergii* var. *minor*	*rbcL* and ITS2	No	-	-	1	-	-	1	1.0%
Hypericaceae	*Hypericum senanense* subsp. *mutiloides*									
Juncaceae	*Juncus filiformis*	*rbcL* and ITS2	No	-	1	7	-	-	8	7.6%
Lentibulariaceae	*Pinguicula vulgaris* var. *macroceras*									
Melanthiaceae	*Helonias orientalis*									
	*Veratrum stamineum* var. *stamineum*									
Menyanthaceae	*Nephrophyllidium crista-galli* subsp. *japonicum*	*rbcL* and ITS2	No	3	7	1	1	-	12	11.4%
Nartheciaceae	*Aletris foliata*	*rbcL*	No	1	-	-	-	-	1	1.0%
	*Narthecium asiaticum*	*rbcL*	No	-	-	1	-	-	1	1.0%
Orchidaceae	*Dactylorhiza aristata*	*rbcL*	No	-	1	3	-	-	4	3.8%
	*Platanthera tipuloides* subsp. *nipponica*									
Orobanchaceae	*Pedicularis chamissonis* var. *japonica*									
	*Pedicularis yezoensis*	*rbcL* and ITS2	No	2	1	-	-	-	3	2.9%
Pinaceae	*Abies mariesii*									
	*Pinus pumila*	*rbcL* and ITS2	Yes	1	-	-	-	-	1	1.0%
Plantaginaceae	*Veronica nipponica*									
Poaceae	*Bromus* sp. (*B*. *inermis*, *B*. *pacificus*)	*rbcL*	No	-	-	8	-	1	9	8.6%
	*Calamagrostis longiseta*	*rbcL*	No	-	-	1	-	-	1	1.0%
	*Moliniopsis japonica*									
	*Sasa kurilensis*	*rbcL*	No	1	1	13	-	-	15	14.3%
Polygonaceae	*Persicaria weyrichii* var. *weyrichii*	*rbcL* and ITS2	No	1	-	-	-	-	1	1.0%
	*Rumex alpestris* subsp. *lapponicus*									
Polytrichaceae	*Polytrichum juniperinum*	ITS2	No	5	3	4	-	-	12	11.4%
Ranunculaceae	*Aconitum hakusanense*	ITS2	No	-	-	1	-	-	1	1.0%
	*Anemone narcissiflora* subsp. *nipponica*	ITS2	Yes	1	-	-	-	-	1	1.0%
	*Coptis trifoliolata*	*rbcL*	No	-	-	2	-	-	2	1.9%
	*Ranunculus acris* subsp. *nipponicus*		Yes							
Rosaceae	*Aruncus dioicus* var. *kamtschaticus*									
	*Potentilla matsumurae*	*rbcL* and ITS2	Yes	2	1	1	2	-	6	5.7%
	*Rubus vernus*	*rbcL*	No	-	-	-	-	1	1	1.0%
	*Sieversia pentapetala*	*rbcL* and ITS2	Yes	17	1	9	2	2	31	29.5%
	*Sorbus commixta*	*rbcL* and ITS2	No	-	-	13	2	1	16	15.2%
Sapindaceae	*Acer tschonoskii*									
Tofieldiaceae	*Triantha japonica*	*rbcL* and ITS2	No	-	5	1	-	-	6	5.7%
Xanthorrhoeaceae	*Hemerocallis dumortieri* var. *esculenta*									
Total number of food plant taxa				151	84	181	64	46	526	

1) Refer to Kobayashi et al., [16], Chiba et al., [17], and Satomi et al., [18].

**Table 5:** Estimated asymptotic Shannon diversity and coverage of food plant candidates for each fecal sampling period.

Feces collection period	No. of fecal samples	No. of identified plant taxa	S_obs_	S_est_	Coverage of food plant candidates (%)
July 8–10, 2016	31	31	16.6	20.1	90.9
July 26–28, 2016	18	23	15.2	21.1	87.0
August 18–20, 2015	31	36	20.6	25.7	90.7
September 13–14, 2018	17	15	11.6	12.9	97.5
October 11–12, 2016	8	16	12.1	14.4	89.5
Total	105	53	23.1	25.0	96.4

S_obs_: Observed Shannon diversity.

S_est_: Estimated asymptotic Shannon diversity.

## Discussion

### Effectiveness of DNA metabarcoding

In this study, a total of 53 plant taxa were identified using a combination of the local database and NCBI searches for *rbcL* and ITS2 sequences for plants found in fecal samples collected in the study area. Of the 53 plant taxa, 49 could be assigned to species, three to genera, and one to family. Further, rarefaction analysis of each fecal sample collection period showed that 96.4% of food plant taxa in the study area were covered throughout the entire study period. In addition, of the 53 plant taxa identified in this study, 32 species and two taxa (a member of family Asteraceae (i.e., *C*. *otayae*, *H*. *japonicum*, or *S*. *virgaurea* subsp. *asiatica*), and a member of genus *Bromus* (either *B*. *inermis* or *B*. *pacificus*) which had not been reported previously as a food plant from July to October were identified for the first time in our study [16–18] (Table 4). ITS2 is generally considered to be more accurate in studies of diet identification by DNA barcoding than chloroplast DNA [42,43] because ITS2 has higher interspecific variation than *rbcL* [41]. However, in the 74 alpine plant species used to construct the local database in this study, the percentage of interspecific DNA sequence variation did not differ between *rbcL* (89.2%) and ITS2 (88.9%). Moreover, by combining the local database and the NCBI database, the number of food plant resources identified by *rbcL* (40 taxa) was higher than that identified by ITS2 (33 taxa) (Table 3); however, ITS2 was used to supplement the plants that could not be identified by *rbcL* (Table 3). Therefore, by combining the local database and NCBI searches for both the *rbcL* and the ITS2 regions, the accuracy of plant species identification was higher than that obtained using only the local database or only NCBI searches (Table 3). In addition, the combined use of the *rbcL* and the ITS2 sequences in the local database improved both the accuracy of plant species identification and the number of plant taxa identified compared to using either of the databases for each region alone.

Comparing our present results with those of previous studies, in a study of the gastric contents of the Japanese rock ptarmigan, 17 plant species were identified from 39 individuals in July to October 1926–1928 [17], and only five plant species were observed in the gastric contents of an adult male in October 1967 [18]. Japanese rock ptarmigans inhabiting Mt. Norikura of Japan’s Northern Alps were observed to forage on 34 plant taxa by direct observation of foraging behavior over 43 days from July to October 2009 [16]. Our previous results obtained using a cloning method and a combination of the local *rbcL* database and NCBI searches of the same *rbcL* DNA sequences used in the present study identified a total of 26 taxa; 22 were identified to species, two to genera, and two to family [22]. Our present study was thus capable of identifying more plant species than was possible by observation of the gastric contents alone [17,18], direct observation of foraging behavior [16], and the cloning method employed in our previous study using the local database and NCBI searches for the same *rbcL* DNA sequences used in the present study [22].

### Characteristics of food resources for Japanese rock ptarmigans

Across all collection periods, the most important food plants for the Japanese rock ptarmigan in this study area belonged to the family Ericaceae (99.0% of 105 fecal samples), especially *E*. *nigrum* var. *japonicum* (68.6%) which was one of the dominant food plants; these findings were consistent with the findings of a previous study conducted on Japanese rock ptarmigans elsewhere in Japan [16]. However, *V*. *ovalifolium* var. *ovalifolium* (69.5%) and *K*. *procumbens* (42.9%), which were frequently identified plant species in the Ericaceae family in this study, were not dominant species in a previous study [16]. These members of the family Ericaceae were dominant species in the community of alpine windswept dwarf shrubs in this study area. From May to June, Japanese rock ptarmigans forage mainly on alpine windswept dwarf shrubs, which have the earliest snowmelt in this study area. After July, the Japanese rock ptarmigans forage not only on alpine windswept dwarf shrubs but also on the community of snow-melted alpine leeward dwarf shrubs [4]. During our study, Japanese rock ptarmigans were observed in both communities, but they mainly foraged on windswept alpine dwarf shrubs. The dominant species of food plants in the study area, such as *E*. *nigrum* var. *japonicum* (68.6%), and subdominant plants, such as *S*. *pentapetala* (29.5%), *P*. *aleutica* (13.3%), *V*. *vitis-idaea* (13.3%), and *B*. *ermanii* (11.4%) were generally the same as those reported in a previous study conducted on Japanese rock ptarmigan in Japan [16]; however, other food plants were endemic to this study area (Table 4). Although *S*. *kurilensis* (14.3%), *N*. *crista-galli* subsp. *japonicum* (11.4%), and *P*. *juniperinum* (11.4%), which were subdominant food plant species in the study area, are common in other habitats elsewhere in Japan, there are no records of these species being utilized by Japanese rock ptarmigans in previous studies (to our knowledge) [16–18]. A possible reason for this absence of foraging records might be the shoot height of these plants being close to the ground, which would make it difficult to accurately observe the birds foraging, but the exact reasons are unknown. The minor food plants in this study area (i.e., less than 10% of 105 fecal samples) have not been reported previously [16–18]; plants with low densities in the study area (*e*.*g*., *Ixeridium dentatum* subsp. *kimuranum*, 7.6%; *Triantha japonica*, 5.7%), plants scattered among other plants (*e*.*g*., *Cornus canadensis*, 5.7%; *Gaultheria adenothrix*, 3.8%; *G*. *pyroloides*, 3.8%; *Coptis trifoliolata*, 1.9%), and plants that are difficult to identify based on their external morphology (*e*.*g*., *Bromus* sp., 8.6%; *Juniperus communis* var. *hondoensis*, 1.9%; *Calamagrostis longiseta*, 1.0%) can easily be overlooked in direct observation studies (Table 4). Moreover, hygrophytes (*e*.*g*., *N*. *crista-galli* subsp. *japonicum*, 11.4%; *J*. *filiformis*, 7.6%) growing in and around small pools were identified for the first time in our study [16–18] (Table 4), indicating that hygrophytes are also an important food resource for Japanese rock ptarmigans in the study area. In addition, *Bromus* sp. (8.6%) and *C*. *longiseta* (1.0%) in family Poaceae (Table 4), which are not native plants in the alpine meadow zone, and might have been imported by mountain climbers, were also an important food plant resource for Japanese rock ptarmigans in this study area.

On the other hand, *Arctous alpina* [17], *Arnica unalaschcensis* var. *tschonoskyi* [16], *Maianthemum dilatatum* [17], *Pedicularis chamissonis* var. *japonica* [16], and *Ranunculus acris* subsp. *nipponicus* [17], all of which were recorded as being foraged by other ptarmigan populations in previous studies, were also observed to grow in this study area; however, none of these plants were detected in any of the fecal samples collected in this study (Table 4). The primer sets for *rbcL* and ITS2 used in this study are universal primers capable of amplifying *rbcL* and ITS2 regions in most plant species [39,50]. In addition, both primer sets were able to amplify both *rbcL* and ITS of *A*. *alpina*, *A*. *unalaschcensis* var. *tschonoskyi*, *M*. *dilatatum*, *P*. *chamissonis* var. *japonica*, and *R*. *acris* subsp. *nipponicus* in the construction of our local database, except for the ITS2 sequence of *M*. *dilatatum*. Therefore, it is not expected that amplification of either *rbcL* or ITS2 will be affected by sequence variations in the regions corresponding to the primers in undetected plant species. Based on these considerations, it is reasonable to assume that these undetected plant species were not foraged by Japanese rock ptarmigans in this study area, but the reason for this is not known. In addition, *Vaccinium shikokianum*, *V*. *smallii* var. *smallii*, and *V*. *uliginosum* var. *japonicum* were also recorded as food plants in other populations in previous studies [16–18] and grow in this study area. We were unable to identify these three *Vaccinium* species at the species level in this study, as these three species have the same *rbcL* and ITS2 sequences. Therefore, it is necessary to select a region other than the *rbcL* and ITS2 regions to identify such food plants. Of the subdominant food plants (more than 10% to less than 30% of the 105 fecal samples), *S*. *pentapetala* was relatively abundant in July, and *S*. *commixta* and *S*. *kurilensis* were relatively abundant in August, compared to the other sampling periods (Fig 3 and Table 4). Since *S*. *pentapetala* and *S*. *commixta* are in full bloom and *S*. *kurilensis* is developing new leaves during this time, Japanese rock ptarmigans are thought to be selectively feeding on the conspicuous flowers and soft new leaves. Given that the DNA metabarcoding method cannot identify specific parts of the food plants, direct observation during fecal sample collection can assist in clarifying which plant parts are utilized by ptarmigan and how this is affected by phenological changes in alpine vegetation. In general, *P*. *pumila* can be found in all Japanese rock ptarmigan habitats in Japan; however, different areas are characterized by their own unique alpine vegetation, *e*.*g*., *E*. *nigrum* var. *japonicum* is a dominant species in ptarmigan habitat in the Northern Japanese Alps, but it is rare in Japanese rock ptarmigan habitat in the Southern Japanese Alps [4]. The major food plant species for the Japanese rock ptarmigan may differ among mountain regions [4]. Therefore, effective conservation of the Japanese rock ptarmigan requires us to clarify the relationship between the flora and food plant resources in different mountain areas, and to develop vegetation conservation measures for the flora of individual mountain regions.

## Conclusions

In this study area, the dominant taxa of the Ericaceae family in the windswept alpine dwarf shrub community were found to be the major food plant for the Japanese rock ptarmigan from July to October, suggesting the need to manage this floral community to conserve ptarmigan food resources. In addition, the local databases constructed in this study can be used to survey other areas with similar flora. The Japanese rock ptarmigan does not fear people, which allows researchers to collect fecal samples efficiently and limits the possibility of mistaking ptarmigan fecal samples for those of other birds. The DNA barcoding method employed in this study is therefore considered to represent an important advance in our ability to elucidate the diet of the Japanese rock ptarmigan, and can also be used as a tool for conserving the alpine plant species that are their food sources.

## Supporting information

S1 Fig Comparison of number of plant taxa per fecal sample identified by querying local and NCBI databases for *rbcL* sequences, local and NCBI queries for ITS2 sequences, and queries using combined *rbcL* and ITS2 local database as well as NCBI results. The numbers of plant taxa per fecal sample identified by the *rbcL* local database, ITS2 local database, and a combination of the *rbcL* and ITS2 local databases are presented as boxplots. Each box delimits values between 25% and 75% of the group. The bold horizontal line represents the median of the group. Whiskers are drawn for obtained values that differ least from the median ± 1.5 interquartile ranges. Different letters indicate statistically significant differences (Steel-Dwass test, P<0.05) between groups.
(PPTX)

S1 Table List of PCR primers. 1) List of initial PCR primers. Italic characters indicate the MiSeq sequencing primers. Bold Ns indicate random bases used to improve the quality of MiSeq sequencing [48]. Single underlined characters indicate DNA barcoding primer sequences. 2) List of second PCR primers. Italic characters indicate the MiSeq sequencing primers. Bold Ns indicate random bases used to improve the quality of MiSeq sequencing [48]. Bold Xs indicate index sequences used to identify each sample. Single underlined characters indicate P5/P7 adapter sequences for MiSeq sequencing and double underlined characters indicate DNA barcoding primer sequences.
(XLSX)

S2 Table Sequencing statistics of *rbcL* and ITS2 regions. Input reads: number of reads in raw fastq files. Filtered reads: number of reads after preliminary quality filtering. DenoisedF/R reads: number of reads after quality filterin. Merged reads: number of merged forward-reverse reads. Nonchim reads: number of merged reads after removal of chimeric sequences.
(XLSX)

S3 Table Homology search results using blastn of each fecal sample using the local *rbcL* local database and NCBI. (XLSX)

S4 Table Homology search results using blastn of each fecal sample using the local ITS2 database and NCBI. (XLSX)

S5 Table Homology search results using blastn on each fecal sample in the *rbcL* region. 1) Local database. 2) NCBI database. 3) Combination of the NCBI and the local database.
(XLSX)

S6 Table Homology search results using blastn on each fecal sample in the ITS2 region. 1) Local database. 2) NCBI database. 3) Combination of the NCBI and the local database.
(XLSX)

S7 Table Homology search results using blastn on each fecal sample when combining the *rbcL* and ITS2 regions. 1) Local database. 2) NCBI database. 3) Combination of the NCBI and the local database.
(XLSX)

S8 Table Food plant families identified using DNA metabarcoding with *rbcL* and ITS2. (XLSX)

## References

[1] Dirnbock T, Essl F, Rabitsch W. Disproportional risk for habitat loss of high‐altitude endemic species under climate change. Global Change Biology. 2011;17(2):990–6.

[2] Sekercioglu CH, Schneider SH, Fay JP, Loarie SR. Climate change, elevational range shifts, and bird extinctions. Conservation biology. 2008;22(1):140–50. doi: 10.1111/j.1523-1739.2007.00852.x 18254859

[3] Theurillat JP, Guisan A. Potential impact of climate change on vegetation in the European alps: a review. Climatic Change. 2001;50:77–109.

[4] Nakamura H. Rock ptarmigan *Lagopus mutus japonicus*. Jpn J Ornithol. 2007;56:93–114 (in Japanese with English summary). doi: 10.1292/jvms.69.171 17339762

[5] Baba Y, Fujimaki Y, Yoshii R, Koike H. Genetic variability in the mitochondrial control region of the Japanese rock ptarmigan *Lagopus mutus japonicus*. Jap. J. Ornithol. 2001;50(2):53–64,107 (in Japanese with English summary).

[6] Hotta M, Tsuyama I, Nakao K, Ozeki M, Higa M, Kominami Y, et al. Modeling future wildlife habitat suitability: serious climate change impacts on the potential distribution of the rock ptarmigan *Lagopus muta japonica* in Japan’s northern Alps. BMC ecology. 2019;19(1):1–14. doi: 10.1186/s12898-018-0217-5 31288795 PMC6617707

[7] Yoshida M, Yoshida M. Vegetations as nests of Japanese ptarmigan, *Lagopus mutus japonicus*, at Murododaira on the Tateyama Mountains. Bull. Bot. Gard. Toyama. 2004;9:23–34 (in Japanese with English summary).

[8] Kobayashi A, Nakamura H. Seasonal changes in the flock composition and altitudinal range of the rock ptarmigan *Lagopus muta japonica* in Japan. Jpn J Ornithol. 2018;67(1):69–86 (in Japanese with English summary).

[9] Haneda K, Nakamura H, Koiwai A, Iizawa T, Tajima K. Distribution and density of the rock ptarmigan *Lagopus* mutus in the Southern Japan Alps. Tori. 1985;34(2–3):33–48 (in Japanese with an English summary).

[10] Suzuki A, Kobayashi A, Nakamura H, Takasu F. Population viability analysis of the Japanese rock ptarmigan *Lagopus muta japonica* in Japan. Wildlife biol. 2013;19(4):339–346.

[11] Kobayashi A. Managing impacts of human trampling along trails in alpine zone. J Japan Inst. Landsc. Archt. 1997;61(5):653–8 (in Japanese with English summary).

[12] Disturbance Kawano S. and conservation of the subalpine−alpine vegetation and biota in the Tateyama−Kurobe National Park, the Japan North Alps in Central Honshu, Japan−The results of long−term monitoring. Jap J Ecol. 1999;49:313–320 (in Japanese with English summary).

[13] Kudo G, Kawai Y, Amagai Y, Winkler DE. Degradation and recovery of an alpine plant community: experimental removal of an encroaching dwarf bamboo. Alp Bot. 2017;127(1):75–83.

[14] Ministry of the Environment. National Conservation Program for Japanese rock ptarmigans. 2014 [cited 2021 April 25]. Available from: https://www.env.go.jp/press/files/jp/24426.pdf (in Japanese).

[15] Ministry of the Environment. National Conservation Program for Japanese rock ptarmigans. 2020 [cited 2021 April 25]. Available from: http://chubu.env.go.jp/shinetsu/raicho-jigyoukeikaku2ki.pdf (in Japanese).

[16] Kobayashi A, Nakamura H. Seasonal change of food items of the Japanese Rock Ptarmigan. Jap. J. Ornithol. 2011;60:200–215 (in Japanese with English summary).

[17] Chiba S. Food analysis of the Japanese ptarmigan. J Yamashina Inst. Ornithol. 1965;4(3–4):184–197 (in Japanese with English summary).

[18] Satomi N, Yuasa S. Food plants of Japanese ptarmigan. The Journal of Geobotany. 1968;16(3):84–90 (in Japanese).

[19] García-González R, Aldezabal A, Laskurain NA, Margalida A, Novoa C. Factors affecting diet variation in the Pyrenean rock ptarmigan (*Lagopus muta pyrenaica*): conservation implications. PLoS One. 2016;11(2): e0148614. doi: 10.1371/journal.pone.0148614 26863532 PMC4749312

[20] García-González R, Aldezabal A, Laskurain NA, Margalida A, Novoa C. Influence of snowmelt timing on the diet quality of Pyrenean rock ptarmigan (*Lagopus muta pyrenaica*): implications for reproductive success. PLoS One. 2016;11(2): e0148632. doi: 10.1371/journal.pone.0148632 26849356 PMC4746074

[21] Fujii T, Ueno K, Minami M. Identification of food plants in the diet of Japanese ptarmigan (*Lagopus mutus japonicus*) in the Japan’s Northern Japan Alps using DNA Barcoding. Wildlife and Human Society. 2019;6(2):13–18.

[22] Fujii T, Ueno K, Minami M. Plant-derived food resources of Japanese rock ptarmigan (*Lagopus muta japonica*) identified by DNA barcoding using *rbcL* local database constructed from alpine plants found in the Northern Japan Alps, Japan. Grouse News. 2019;57:13–19.

[23] Ando H, Setsuko S, Horikoshi K, Suzuki H, Umehara S, Inoue‐Murayama M, et al. Diet analysis by next‐generation sequencing indicates the frequent consumption of introduced plants by the critically endangered red‐headed wood pigeon (*Columba janthina nitens*) in oceanic island habitats. Ecol. Evol. 2013;3(12):4057–4069. doi: 10.1002/ece3.773 24324859 PMC3853553

[24] Aziz SA, Clements GR, Peng LY, Campos-Arceiz A, McConkey KR, Forget PM, et al. Elucidating the diet of the island flying fox (*Pteropus hypomelanus*) in Peninsular Malaysia through Illumina Next-Generation Sequencing. PeerJ. 2017;5:e3176. doi: 10.7717/peerj.3176 28413729 PMC5391789

[25] Deagle BE, Kirkwood R, Jarman SN. Analysis of Australian fur seal diet by pyrosequencing prey DNA in faeces. Mol. Ecol. 2009;18(9):2022–2038. doi: 10.1111/j.1365-294X.2009.04158.x 19317847

[26] Lim VC, Ramli R, Bhassu S, Wilson JJ. Pollination implications of the diverse diet of tropical nectar-feeding bats roosting in an urban cave. PeerJ. 2018;6:e4572. doi: 10.7717/peerj.4572 29607265 PMC5875395

[27] Lopes CM, De Barba M, Boyer F, Mercier C, Da Silva Filho PJS, Heidtmann LM, et al. DNA metabarcoding diet analysis for species with parapatric vs sympatric distribution: a case study on subterranean rodents. Heredity. 2015;114(5):525–536. doi: 10.1038/hdy.2014.109 25649502 PMC4815513

[28] Mollot G, Duyck P-F, Lefeuvre P, Lescourret F, Martin J-F, Piry S, et al. Cover cropping alters the diet of arthropods in a banana plantation: a metabarcoding approach. PloS one. 2014;9(4):e93740. doi: 10.1371/journal.pone.0093740 24695585 PMC3973587

[29] Rytkönen S, Vesterinen EJ, Westerduin C, Leviäkangas T, Vatka E, Mutanen M, et al. From feces to data: A metabarcoding method for analyzing consumed and available prey in a bird‐insect food web. Ecology and evolution. 2019;9(1):631–9. doi: 10.1002/ece3.4787 30680143 PMC6342092

[30] Sato JJ, Shimada T, Kyogoku D, Komura T, Uemura S, Saitoh T, et al. Dietary niche partitioning between sympatric wood mouse species (Muridae: *Apodemus*) revealed by DNA meta-barcoding analysis. J. Mammal. 2018;99(4):952–964.

[31] Shirako T, Ishizawa Y, Ajioka Y, Aichi M, Ueno K, Hai BT, et al. Identification of Muridae species and their food resources using DNA barcoding in Cat Tien National Park, Vietnam. Mammal study. 2015;40(4):217–229.

[32] Soininen EM, Valentini A, Coissac E, Miquel C, Gielly L, Brochmann C, et al. Analysing diet of small herbivores: the efficiency of DNA barcoding coupled with high-throughput pyrosequencing for deciphering the composition of complex plant mixtures. Front. Zool. 2009;6(1):16. doi: 10.1186/1742-9994-6-16 19695081 PMC2736939

[33] Soininen EM, Ravolainen VT, Bråthen KA, Yoccoz NG, Gielly L, Ims RA. Arctic small rodents have diverse diets and flexible food selection. PLoS One. 2013;8(6): e68128. doi: 10.1371/journal.pone.0068128 23826371 PMC3694920

[34] Soininen EM, Zinger L, Gielly L, Bellemain E, Bråthen KA, Brochmann C, et al. Shedding new light on the diet of Norwegian lemmings: DNA metabarcoding of stomach content. Polar Biol. 2013;36(7):1069–1076.

[35] Sullins DS, Haukos DA, Craine JM, Lautenbach JM, Robinson SG, Lautenbach JD, et al. Identifying the diet of a declining prairie grouse using DNA metabarcoding. The Auk: Ornithological Advances. 2018;135(3):583–608.

[36] Vesterinen EJ, Lilley T, Laine VN, Wahlberg N. Next generation sequencing of fecal DNA reveals the dietary diversity of the widespread insectivorous predator Daubenton’s bat (*Myotis daubentonii*) in Southwestern Finland. PLoS One. 2013;8(11): e82168. doi: 10.1371/journal.pone.0082168 24312405 PMC3842304

[37] Yang Y, Zhan A, Cao L, Meng F, Xu W. Selection of a marker gene to construct a reference library for wetland plants, and the application of metabarcoding to analyze the diet of wintering herbivorous waterbirds. PeerJ. 2016;4:e2345. doi: 10.7717/peerj.2345 27602302 PMC4991844

[38] Little DP. A DNA mini‐barcode for land plants. Molecular Ecology Resources. 2014;14(3):437–46. doi: 10.1111/1755-0998.12194 24286499

[39] Cheng T, Xu C, Lei L, Li C, Zhang Y, Zhou S. Barcoding the kingdom Plantae: new PCR primers for ITS regions of plants with improved universality and specificity. Mol. Ecol. Resour. 2016;16(1):138–149. doi: 10.1111/1755-0998.12438 26084789

[40] Fahner NA, Shokralla S, Baird DJ, Hajibabaei M. Large-scale monitoring of plants through environmental DNA metabarcoding of soil: recovery, resolution, and annotation of four DNA markers. PloS one. 2016;11(6):e0157505. doi: 10.1371/journal.pone.0157505 27310720 PMC4911152

[41] Weihong B, Li D, Li X. DNA barcoding of *Actinidia* (Actinidiaceae) using internal transcribed spacer, *matK*, *rbcL* and *trnH-psbA*, and its taxonomic implication. N. Z. J. Bot. 2018;56(4):360–371.

[42] Kim W, Ji Y, Choi G, Kang Y, Yang S, Moon B. Molecular identification and phylogenetic analysis of important medicinal plant species in genus Paeonia based on rDNA-ITS, *matK*, and *rbcL* DNA barcode sequences. Genet Mol Res. 2016;15(3): gmr.15038472. 10.4238/gmr.15038472 27525917

[43] Staats M, Arulandhu AJ, Gravendeel B, Holst-Jensen A, Scholtens I, Peelen T, et al. Advances in DNA metabarcoding for food and wildlife forensic species identification. Analytical and Bioanalytical Chemistry. 2016;408(17):4615–4630. doi: 10.1007/s00216-016-9595-8 27178552 PMC4909793

[44] Chua PY, Crampton‐Platt A, Lammers Y, Alsos IG, Boessenkool S, Bohmann K. Metagenomics: A viable tool for reconstructing herbivore diet. Molecular Ecology Resources. 2021; 21:2249–2263. doi: 10.1111/1755-0998.13425 33971086 PMC8518049

[45] Nakahama N, Furuta T, Ando H, Setsuko S, Takayanagi A, Isagi Y. DNA meta-barcoding revealed that sika deer foraging strategies vary with season in a forest with degraded understory vegetation. Forest Ecology and Management. 2021;484:118637.

[46] Anthony SJ, Islam A, Johnson C, Navarrete-Macias I, Liang E, Jain K, et al. Non-random patterns in viral diversity. Nat. Commun. 2015;6(1):1–7. doi: 10.1038/ncomms9147 26391192 PMC4595600

[47] Bonillaa JS, Zambrana-Torrelioc C, Rubiod EL, Aguirree AA. Viral diversity of bat communities in human-dominated landscapes in Mexico. Vet. Méx OA. 2015;2(1):1–23.

[48] Chao A, Gotelli NJ, Hsieh T, Sander EL, Ma K, Colwell RK, et al. Rarefaction and extrapolation with Hill numbers: a framework for sampling and estimation in species diversity studies. Ecol. Monogr. 2014;84(1):45–67.

[49] Hsieh T, Ma K, Chao A. iNEXT: an R package for rarefaction and extrapolation of species diversity (Hill numbers). Methods Ecol. Evol. 2016;7(12):1451–1456.

[50] Matsuki R, Shimano K, Abe S, Yatake H, Takeuchi T, Shiraki S, et al. Study on the ecosystem sustaining a pair of Golden Eagles -Identification of food plants by DNA analysis from animal feces-. CRIEPI Research Report. 2003:U03008 (in Japanese with English summary).

[51] Lundberg DS, Yourstone S, Mieczkowski P, Jones CD, Dangl JL. Practical innovations for high-throughput amplicon sequencing. Nat. Methods. 2013;10(10):999–1002. doi: 10.1038/nmeth.2634 23995388

[52] Camacho C, Coulouris G, Avagyan V, Ma N, Papadopoulos J, Bealer K, et al. BLAST+: architecture and applications. BMC bioinformatics. 2009;10(1):1–9. doi: 10.1186/1471-2105-10-421 20003500 PMC2803857

[53] Toju H, Tanabe A, Ishii H. Ericaceous plant–fungus network in a harsh alpine–subalpine environment. Mol. Ecol. 2016;25(13):3242–3257. doi: 10.1111/mec.13680 27136380

[54] Tanabe AS, Toju H. Two new computational methods for universal DNA barcoding: a benchmark using barcode sequences of bacteria, archaea, animals, fungi, and land plants. PLoS One. 2013;8(10):e76910. doi: 10.1371/journal.pone.0076910 24204702 PMC3799923

[55] Callahan BJ, McMurdie PJ, Rosen MJ, Han AW, Johnson AJA, Holmes SP. DADA2: high-resolution sample inference from Illumina amplicon data. Nat. Methods. 2016;13(7):581–583. doi: 10.1038/nmeth.3869 27214047 PMC4927377

[56] R Core Team. R: a language and environment for statistical computing (version 3.5. 3) [software]. 2019.

[57] Oksanen J, Blanchet F, Friendly M, Kindt R, Legendre P, McGlinn D, et al. vegan: Community Ecology Package. R package version 2.5–5; 2019.

[58] Iwatsuki K, Yamazaki T, Boufford DE, Ohba H. Flora of Japan Volume I. Kodansha Tokyo Press; 1995.

[59] Iwatsuki K, Boufford DE, Ohba H. Flora of Japan Volume IIa. Kodansha Tokyo Press; 2006.

[60] Iwatsuki K, Boufford DE, Ohba H. Flora of Japan Volume IIb. Kodansha Tokyo Press; 2001.

[61] Iwatsuki K, Boufford DE, Ohba H. Flora of Japan Volume IIc. Kodansha Tokyo Press; 1999.

[62] Iwatsuki K, Yamazaki T, Boufford DE, Ohba H. Flora of Japan Volume IIIa. Kodansha Tokyo Press; 1993.

[63] Iwatsuki K, Yamazaki T, Boufford DE, Ohba H. Flora of Japan Volume IIIb. Kodansha Tokyo Press; 1995.

[64] Iwatsuki K, Boufford DE, Ohba H. Flora of Japan Volume Iva. Kodansha Tokyo Press; 2020.

[65] Iwatsuki K, Boufford DE, Ohba H. Flora of Japan Volume IVb. Kodansha Tokyo Press; 2020.

[66] Yoshida M, Yamashita T. Changes of the species composition in “specific plant communities” in Toyama in the last 30 years. Bull. Bot. Grad. Toyama. 2008;13:1–14.

[67] Schneider G, Chicken E, Becvarik R. NSM3: Functions and Datasets to Accompany Hollander, Wolfe, and Chicken—Nonparametric Statistical Methods, Third Edition, R package version 1.16; 2021.

[68] Chao A, Colwell RK, Lin C-W, Gotelli NJ. Sufficient sampling for asymptotic minimum species richness estimators. Ecology. 2009;90(4):1125–1133. doi: 10.1890/07-2147.1 19449706

