# Supplementary material for: Identification of Lagopus muta japonica food plant resources in the Northern Japan Alps using DNA metabarcoding
Source: PLoS One. 2022 Mar 10;17(3):e0252632. doi: 10.1371/journal.pone.0252632 (PMC8912148; doi:10.1371/journal.pone.0252632)
Supplement: S1 Fig — The numbers of plant taxa per fecal sample identified by the rbcL local database, ITS2 local database, and a combination of the rbcL and ITS2 local databases are presented as boxplots. Each box delimits values between 25% and 75% of the group. The bold horizontal line represents the median of the group. Whiskers are drawn for obtained values that differ least from the median ± 1.5 interquartile ranges. Different letters indicate statistically significant differences (Steel-Dwass test, P<0.05) between groups. (PPTX) [file pone.0252632.s001.pptx]

## Slide 1
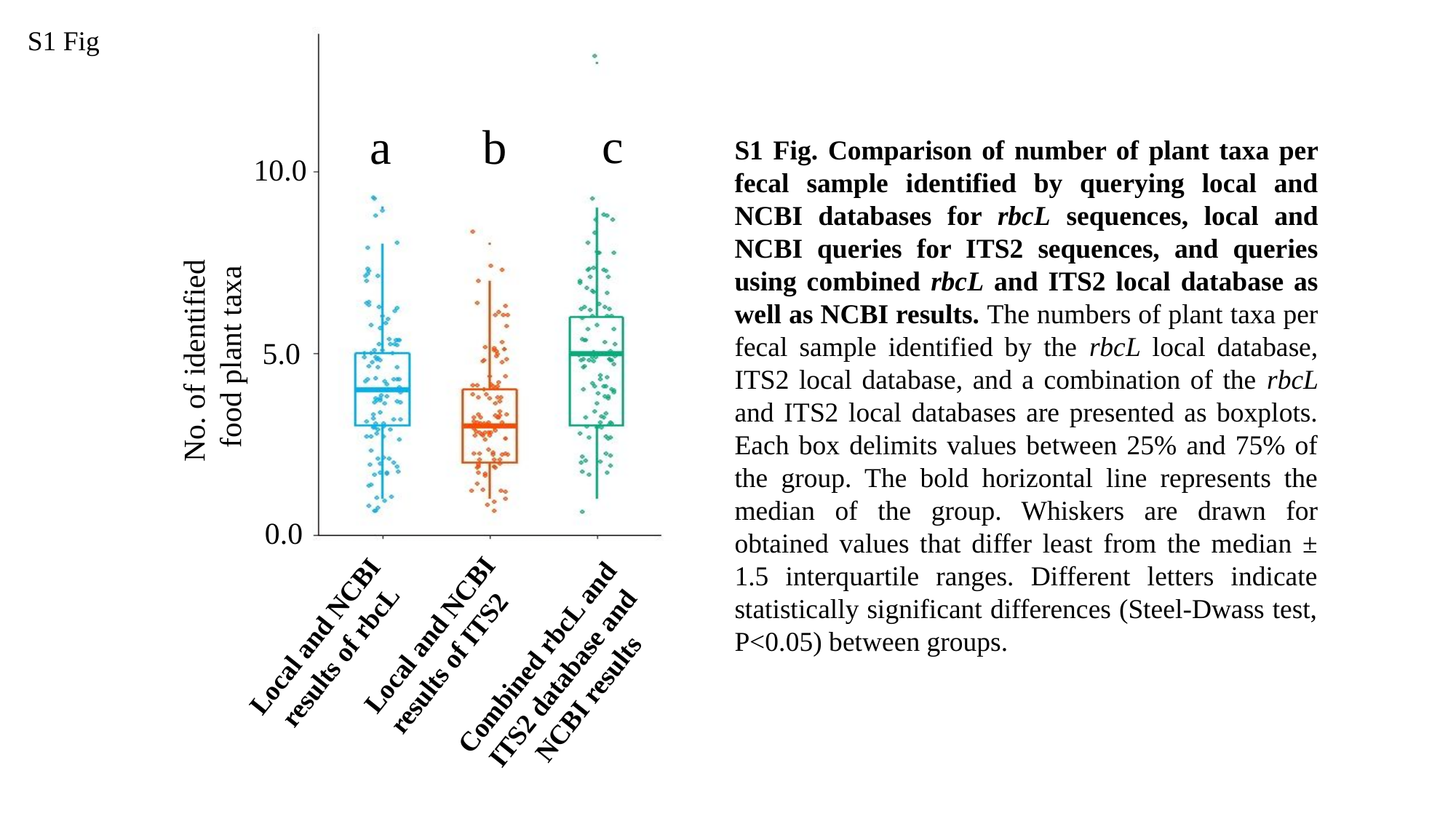

S1 Fig
c
a
b
10.0
No. of identified
food plant taxa
5.0
0.0
Local and NCBI
results of ITS2
 Local and NCBI
results of rbcL
Combined rbcL and ITS2 database and NCBI results
S1 Fig. Comparison of number of plant taxa per fecal sample identified by querying local and NCBI databases for rbcL sequences, local and NCBI queries for ITS2 sequences, and queries using combined rbcL and ITS2 local database as well as NCBI results. The numbers of plant taxa per fecal sample identified by the rbcL local database, ITS2 local database, and a combination of the rbcL and ITS2 local databases are presented as boxplots. Each box delimits values between 25% and 75% of the group. The bold horizontal line represents the median of the group. Whiskers are drawn for obtained values that differ least from the median ± 1.5 interquartile ranges. Different letters indicate statistically significant differences (Steel-Dwass test, P<0.05) between groups.
